# Influence of the COVID-19 Pandemic on Patients with Rectal Cancer

**DOI:** 10.3390/jcm13123568

**Published:** 2024-06-18

**Authors:** Fiona Speichinger, Ann-Kathrin Berg, Ani Stoyanova, Johannes Christian Lauscher, Carsten Kamphues, Katharina Beyer, Claudia Seifarth, Nadia Slavova, Christian Schineis

**Affiliations:** 1 Department of General and Visceral Surgery, Charité Universitätsmedizin Berlin, 12203 Berlin, Germanychristian.schineis@charite.de (C.S.); 2 Department of General and Visceral Surgery, Park-Klinik Weißensee Berlin, 13086 Berlin, Germany

**Keywords:** COVID-19 pandemic, SARS-CoV-2, rectum cancer, lockdown

## Abstract

**Objectives**: The COVID-19 pandemic and its associated restrictions have resulted in delayed diagnoses across various tumor entities, including rectal cancer. Our hypothesis was based on the expectation of a reduced number of primary operations due to higher tumor stages compared to the control group. **Methods**: In a single-center retrospective study conducted from 1 March 2018 to 1 March 2022, we analyzed 120 patients with an initial diagnosis of rectal cancer. Among them, 65 patients were part of the control group (pre-COVID-19), while 55 patients were included in the study group (during the COVID-19 pandemic). We compared tumor stages, treatment methods, and complications, presenting data as absolute numbers or mean values. **Results**: Fewer primary tumor resections during the COVID-19 pandemic (*p* = 0.010), as well as a significantly lower overall number of tumor resections (*p* = 0.025) were seen compared to the control group. Twenty percent of patients in the COVID-19 group received their diagnosis during lockdown periods. These patients presented significantly higher tumor stages (T4b: 27.3% vs. 6.2%, *p* = 0.025) compared to the control group prior to the pandemic. In addition, more patients with angiolymphatic invasion (ALI) were identified in the COVID-19 group following neoadjuvant treatment compared to the control group (*p* = 0.027). No differences were noted between the groups regarding complications, stoma placement, or conversion rates. **Conclusions**: The COVID-19 pandemic, particularly during lockdown, appears to have contributed to delayed diagnoses, resulting in higher tumor stages and a decreased number of surgeries. The quality of rectal cancer treatment can be maintained under pandemic conditions.

## 1. Introduction

The COVID-19 pandemic declared by the WHO in March 2020 led to numerous restrictions in everyday life, including limited access to medical care [1,2]. Especially, concerns about infection and uncertainty among the population led to a reduction in consultations with doctors [3,4] and thus to reduced or changed screening intervals for various types of cancer in general and colorectal cancer in particular, including fecal tests and endoscopy [5,6,7,8,9]. Thus, observations during the pandemic revealed decreased incidence rates of colorectal cancer [10,11,12]. Delaying colonoscopy following a positive fecal test could result in increased mortality rates, as it may lead to the progression to more advanced tumor stages. This also imposes significant economic burdens due to additional healthcare costs [13,14]. This is especially true for rectal cancer since it is one of the leading cancers worldwide [15]. In Germany alone, the incidence of rectal cancer is around 17–27 cases per 100,000 inhabitants [16].

During the COVID-19 pandemic, the number of rectum resections was reduced worldwide [9], including in Germany [17]. Cancer treatment delay results in higher mortality [18]. Early detection is crucial, especially for rectal cancer, particularly in the middle and lower thirds of the rectum. As a low-lying rectal cancer advances, preserving the sphincter becomes increasingly challenging, impacting quality of life [19]. 

Since colon and rectal cancer are essentially different in terms of anatomy, management, and treatment [20], the tumor entities were considered separately. Only a few groups analyzed rectal cancer patients alone [9,21,22,23], and as far as we know, the impact of rectal cancer in terms of pre- and postoperative tumor stage distribution and consecutive therapy has not yet been adequately studied over the entire pandemic period. For colon cancer, it has already been shown that the COVID-19 pandemic had a significant influence on the diagnostic and therapeutic process, with higher rates of more advanced tumor stages, increased emergency operations, and more discontinuity resections [24]. Therefore, we hypothesized that similar patterns may occur regarding delayed diagnoses, more advanced tumor stages, and an increased incidence of emergency surgeries in rectal cancer cases.

## 2. Materials and Methods

We performed a retrospective analysis of 120 consecutive patients with an initial diagnosis of rectum cancer at the Department of General and Visceral Surgery at the Charité University Hospital (Campus Benjamin Franklin, Berlin, Germany) between March 2018 and March 2022. Exclusion criteria were age < 18 years, patients for second opinion, recurrent cancer, lymphomas, cancers at the rectosigmoid junction, and neuroendocrine as well as gastrointestinal stromal tumors. Only rectal cancers diagnosed through endoscopy or cross-sectional imaging (CT and pelvic MRI) and histologically confirmed as adenocancers prior to further therapy were included. The date of the initial diagnosis was decisive for assigning individuals to the two groups. The height of rectal cancers was classified using rigid rectoscopy according to the UICC classification into upper (12–16 cm), middle (6–<12 cm), and lower (<6 cm) third of the rectum. The starting point is by definition the anocutaneous line given as centimeter ab ano (aa). 

The study group comprised all patients diagnosed with rectal cancer during the COVID-19 pandemic (1 March 2020–28 February 2022). The control group consisted of all patients diagnosed during a comparable period preceding the COVID-19 pandemic (01 March 2018–29 February 2020). Of these, 65 patients were assigned to the control group (pre-COVID-19), and 55 patients to the study group (during the COVID-19 pandemic). Medical history and anthropometric parameters were recorded (Table 1). The institutions’ medical ethics committee (Charité Ethics Committee, Universitätsmedizin Berlin) reviewed and approved the protocol and the design of this study (number of proposal: EA4/007/23). All participants gave their written general informed consent upon admission to the hospital. 

Statistical analysis was carried out using SPSS 27 (IBM, Armonk, NY, USA). Interval-scaled data (age and body mass index (BMI)) showed a Gaussian distribution and were therefore presented as mean values with standard deviations. The remaining data were given as absolute numbers. Significance levels were determined using *t*-test for normally distributed interval-scaled data as well as Chi-squared for nominal data. *p*-values < 0.05 were considered significant. 

## 3. Results

### 3.1. Clinical Parameters

This study retrospectively analyzed 120 consecutive patients with an initial diagnosis of rectum cancer. In total, 65 patients were included in the control group (pre-COVID-19) and 55 of them belonged to the study group (during COVID-19 pandemic). Anthropometric parameters including sex and body mass index (BMI) as well as age did not differ between the groups. The height distribution of rectal cancer showed no difference in the distribution. Neoadjuvant therapy (*p* = 0.210) and palliative treatment (*p* = 0.369) were carried out equally frequently in both groups. In the control group, twice as many patients underwent primary tumor resection compared to the study group (23.6% vs. 46.2%, *p* = 0.010). No significant differences were found between the two groups regarding the number of neoadjuvant-treated patients, progress before surgery, ileus, perforation, or chronic inflammatory bowel disease (Table 1).

### 3.2. Characteristics of the Operated Patients

In total, 58 patients in the control group and 43 in the COVID-19 group underwent surgery. Of these, 51 (pre-COVID) and 36 (COVID-19 pandemic) patients underwent tumor resection (*p* = 0.545). In both groups, the majority of patients underwent laparoscopic or robot-assisted surgery (72.4% vs. 81.4%, *p* = 0.294). The conversion rate did not differ between the groups (*p* = 0.851). Furthermore, no significant differences were observed between the groups regarding discontinuity resection or the frequency of stoma creation (*p* = 0.372 and *p* = 0.392, respectively). No significant differences between preoperative morbidity—classified according to the American Society of Anesthesiologists (ASA) [25]—and postoperative complications—classified according to Clavien Dindo [26]—after tumor resection could be found (Table 2). However, there was a trend towards fewer complications in the COVID-19 group (*p* = 0.084). 

### 3.3. Characteristics of Rectal Cancer

According to the 8th American Joint Committee on Cancer (AJCC)/Union Internationale Contre le Cancer (UICC) Tumor-Node-Metastasis (TNM) staging system [27,28,29], Table 3 shows tumor stages at the time of diagnosis as well as after operative/endoscopic tumor resection. In the control group, a significantly higher incidence of UICC IIB was observed at the time of diagnosis compared to the COVID-19 pandemic group (*p* = 0.033). No further significant differences were found (Table 3, Tables S1 and S2). The comparison by local spread diagnostics using MRI [30,31] showed no difference between the groups with regard to extramural vascular infiltration (EMVI) and circumferential resection margin (CRM). Local spread diagnostics using MRI were not available for all patients. The reasons for this were either initial palliative care or contraindications for MRI, e.g., pacemakers, patient refusal, and emergencies (Table 3).

Further pathological findings are listed in Table 4. There were no differences between the control and COVID-19 pandemic group with regard to the degree of differentiation (tumor grading) or the mismatch repair system (MMR).

### 3.4. Characteristics of Neoadjuvant-Treated Patients

Table 5 shows tumor stages of neoadjuvant-treated patients at the time of diagnosis and after tumor resection. A significantly higher number of patients with angiolymphatic invasion could be observed in the COVID-19 group (*p* = 0.027). No further significant differences were found. The postoperative tumor stage could not be determined for all neoadjuvant pre-treated patients, since in the COVID-19 group one patient was transferred to a palliative concept after tumor progression, one patient showed a complete clinical response and was transferred to a watch-and-wait concept, and a total of four patients had the resection performed in external hospitals. In the control group, four patients showed tumor progression and two patients underwent external resection (Table 5 and Table S3).

### 3.5. Subgroup Analysis of COVID-19 Group during Lockdown

To analyze whether an extended lockdown affects the tumor stage at initial diagnosis, we conducted a subgroup analysis within the COVID-19 group. We compared patients in this group who received their initial diagnosis during the lockdown periods with the control group. It was observed that significantly more T4b stages (27.3% vs. 6.2%, *p* = 0.025) were diagnosed with more frequent neoadjuvant therapy (72.7% vs. 43.1%, *p* = 0.069) and fewer primary tumor resections (*p* = 0.021) during the lockdowns compared to the control group (Table 6).

## 4. Discussion

This study provides a complete analysis of patients (operable and non-operable) with an initial diagnosis of rectal cancer during the entire COVID-19 pandemic compared to patients in the same period before COVID-19. Furthermore, we investigated the impact of prolonged lockdown periods on tumor stages at initial diagnosis within the study group.

Regarding the characteristics of the analyzed patients, we saw no significant differences between the COVID-19 pandemic and the control group for age, sex, BMI, tumor localization, or emergencies such as perforation and ileus. No significant differences were found in terms of preoperative morbidity or postoperative complications according to the Clavien Dindo classification [26] for tumor resections. However, there was a trend towards fewer postoperative complications in the COVID-19 group compared to the control group. One explanation for this could be the lower surgical workload in our hospital during the pandemic, i.e., fewer operations and therefore less stress and time pressure. No differences were found with regard to conversion rates, discontinuity resection, or the prevalence of stomata. This demonstrates that consistent surgical quality could be maintained even during the pandemic. This stands in contrast to Dong et al. [21], who found higher postoperative complication rates during the pandemic. Nonetheless, these results cannot be directly compared with ours, as the cohorts differ significantly in terms of inclusion criteria. Dong et al. only investigated patients who received neoadjuvant treatment. Although the present study only refers to the immediate postoperative outcome, other groups did not find any increased mortality in rectal cancer patients treated during the pandemic [32]. 

In our data, we could also demonstrate that the rates of laparoscopic/robotic and open surgeries did not differ. Similar numbers of surgeries were performed minimal invasive and open in both study periods. This corresponds to the data from Fujita et al. [22], who also observed no decline in laparoscopic rectal resection during the pandemic in a retrospective study. This can be explained by the fact that even in cases of nodal positivity or locally advanced rectal cancer, surgical expertise in laparoscopic surgery has advanced to the extent that these entities are no longer contraindications for a laparoscopic operation. However, other studies observed a higher rate of open surgeries [21], which can be explained by the fear of aerosol exposure during laparoscopy [33], as the SARS-CoV-2 virus was detected in the peritoneal fluid [34]. In Germany, there were no recommendations in this regard, so the choice of surgical procedure—before and during the COVID-19 pandemic—was dependent on general indications and contraindications for laparoscopic/robotic versus open surgery.

Overall, we observed fewer rectal-cancer-related operations during the COVID-19 period compared to the control period (78.2 vs. 89.2%). This trend aligns with findings from previous studies [9,35]. Specifically, regarding tumor resections, we noted a significant decrease in total tumor resections during the COVID-19 pandemic, with primary resections halving compared to the control period. This is consistent with the observation by Aparicio et al., who observed an overall decrease in tumor resections of the digestive tract during the pandemic [11]. In addition, our finding mirrors that of Uttinger et al. [17], who also reported a decline in rectal cancer resections during lockdown periods. However, in contrast, a retrospective analysis of medium-sized hospitals in Germany by Hunger et al. [36] did not show a decline in resection rates. Comparability is limited due to the inclusion criteria as this study only analyzed data from non-university hospitals. University hospitals bore the brunt of COVID-19 care, leading them to scale down capacities in certain areas to ensure societal care. In contrast, non-university hospitals often managed regular non-COVID-19 care without capacity restrictions, resulting in many cases of cancers, if not too complex, being surgically treated en masse at these hospitals. Many primary care physicians directly referred patients to smaller, non-university hospitals for surgical care immediately after the initial diagnosis of rectal cancer. This could also account for the trend towards higher tumor stages observed at the initial diagnosis.

Depending on the tumor stage and location, patients received either neoadjuvant therapy followed by resection or primary resection as part of a curative treatment approach. In comparing the two groups, we found no difference in the frequency of neoadjuvant therapy. A Korean group found an increase in neoadjuvant therapy during the COVID-19 pandemic, but only observed a period between March and September 2020 [37]. Fujita et al. [22] detected an increase of neoadjuvant chemoradiotherapy during the COVID-19 pandemic only at the beginning of the pandemic, while Morris et al. [9] observed a significant increase in radiotherapy starting in April 2020. The authors hypothesized that the increase could be a possible ‘bridge to surgery’ [9]. Unfortunately, the observation period does not cover the entire pandemic, specifically only a brief interval of 10 months in 2020 [9], and thus only at the beginning of the COVID-19 pandemic. The differences at the beginning of the pandemic could be due to the fact that the effects of COVID-19 on perioperative outcomes were initially completely unclear and therefore the surgical risk for patients could not be assessed. Later data confirmed increased perioperative morbidity and mortality in COVID-positive patients [38]. Solutions for faster and more accurate diagnostics for SARS-CoV-2 reduced the perioperative risk over the course of the pandemic.

The lack of difference regarding the significantly lower number of primary operations and the statistically insignificant difference in increased neoadjuvant therapy and palliative therapy in our COVID-19 group is likely attributed to the small patient sample size and requires further multicentric analyses.

The comparison of the initial tumor stages between the control and the study group showed no differences, except a significantly higher proportion of the control group for UICC stage IIb (Table 3). We consider this to be a coincidence, and it is in line with a comparable study by Sutton et al. [23], which analyzed rectal cancers over the complete period of the COVID-19 pandemic, but without a differentiation of lockdown times [23].

A closer analysis of our patients with regard to restrictions during the lockdowns showed more initially advanced tumor stages during lockdowns (T4b: 27.3% vs. 6.2%), with a consecutive trend towards a higher proportion of neoadjuvant-treated patients compared to the control group (Table 6). This is consistent with the observations of a Spanish group that found higher rates of stage IV colorectal cancer during the lockdown [39]. Other studies showed similar results at the beginning of the pandemic, comparable to our lockdown periods, with a trend towards higher tumor stages [40,41,42] and fewer early-stage cancers [10] during the COVID-19 pandemic. However, these studies did not distinguish between colon and rectal cancer. One explanation for the fact that more advanced tumor stages only increased significantly during the lockdown phases could be due to the observation that screening decreased significantly, especially at the beginning of the pandemic [43,44], and in particular during lockdown episodes [45]. Furthermore, especially during the lockdown periods, the capacities of large university hospitals were restricted due to the high influx of critically ill COVID-19 patients. As a result, it presumed that many uncomplicated cases were referred to peripheral, smaller hospitals, while only complex, more advanced tumor cases were directly assigned to university hospitals. However, the limitation of this finding lies in the small number of patients diagnosed during lockdown episodes. This, in turn, also indicates that during the lockdown episodes, fewer patients with initial diagnoses of rectal cancer sought medical attention. Only 11 out of 55 patients (20%) in the COVID-19 group received a cancer diagnosis over a cumulative period of more than 7 months. In order to make a generally transferable statement, further patients must be examined more closely during the lockdown periods.

The postoperative TNM classification of all resected rectal cancer patients (Table 3 and Table S2) showed no difference between the control and study groups, as did the analysis of the postoperative stages after neoadjuvant treatment (Table 5 and Table S3). However, a higher proportion of patients with angiolymphatic invasion was observed among those who received neoadjuvant treatment during the COVID-19 pandemic, indicating an increased risk of developing lymph node metastases in the future [46]. A comparable study recorded the postoperative stages of neoadjuvant pre-treated patients with rectal cancer as well and found no difference between the groups [21]. The limiting factors here are the short survey period (January 2019–February 2020) and the inclusion in the COVID-19 group being defined as ‘last radiotherapy 1 September 2019 or later’ [21]. Since the pandemic was only officially declared in early 2020, the patients included in this study received neoadjuvant treatment, and thus, primarily, pre-therapeutic diagnostics, well before the official onset of the pandemic. Therefore, the absolute influence of the pandemic is questionable. Additionally, a Korean study found no difference in postoperative tumor staging between neoadjuvant pre-treated or primary resected patients before and during the COVID-19 pandemic. However, they observed more patients in the COVID-19 group (March–September 2020) with adherent organs to the tumor, as well as higher proportions of angiolymphatic invasion during the pandemic among patients without neoadjuvant treatment [37]. The proportion of rectum cancer patients alone is unclear due to a joint analysis of colon and rectal cancers.

In summary, studies that did not differentiate between the rectum and colon showed more advanced postoperative tumor stages during the COVID-19 pandemic [47,48,49]. The lack of differentiation between colon and rectal cancer makes comparability difficult due to the different treatment regimen, particularly with regard to the often-missing information on neoadjuvant therapy and initial tumor staging (cTNM).

In conclusion, it can be stated that the COVID-19 pandemic appears to have a lesser impact on the diagnosis, treatment, and short-term outcomes of patients suffering from rectal cancer compared to those suffering from colon cancer. The lesser impact of the COVID-19 pandemic on patients with rectal cancer compared to colon cancer could be attributed to earlier and distinct clinical presentations [20]. This could be more frightening for patients compared to the more unspecific symptoms of colon cancer and may outweigh the fear of infection with the SARS-CoV-2 virus.

A limiting factor of our study is the relatively small number of included patients. However, it should be noted that we only included patients diagnosed with rectal cancer during the pandemic. Due to the single-center analysis of a university hospital, the transferability is also limited, but it should be noted that so far, few overall data are available from Europe, especially Germany, in contrast to Asia and the United States. Furthermore, the current study analyzed the entire COVID-19 pandemic and not just sections.

It is also limiting to mention that there is no evaluation of survival times in this study. In order to capture the long-term effects of the pandemic, further analyses are therefore required in the future, particularly of recurrence-free and overall survival.

Another limitation is the informative value of the preoperative stages. On the one hand, not all patients underwent the same imaging, especially with regard to an MRI of the pelvis, and on the other hand, the specificity and sensitivity of the imaging is limited and dependent on the examiner. A pure analysis of pathological stages in turn distorted the data of the present study, due to different pre-treatments and the lack of the possibility of matched groups due to limited patient numbers. In addition, the palliative stages would have been excluded from the analysis, as there is usually no pathological tumor stage due to the lack of surgery. Therefore, in the present study, we provide a complete overview by analyzing the pre- and postoperative stages.

Summarizing our data reveals that large hospitals and university clinics face not only the burden of pandemic management with its associated challenges and strains on infrastructure and staff, but also an increased need to treat more complex tumor stages during pandemics. This, in turn, poses a significant challenge to the infrastructure and capacity of these hospitals. These findings should prompt considerations in preparation for potential future pandemic situations on how these hospitals can be optimally staffed and structured to address such cases effectively.

## 5. Conclusions

The COVID-19 pandemic imposed numerous restrictions on both the medical and private sectors, causing widespread concerns among the population [1]. A noticeable consequence was delayed diagnoses leading to more advanced tumor stages across various cancer types [41,42]. While the pandemic led to a significant impact on colon cancer patients [24], the influence on rectal cancer seems comparatively less pronounced. Differences were mainly observed in the reduced total and primary tumor resections. The shift to more advanced tumor stages was especially evident during lockdown episodes. The effects of the pandemic on tumor progression appeared to vary depending on the specific cancer type. Furthermore, our data demonstrated that the burden on university clinics due to a shift towards more advanced tumor stages, coupled with pandemic management, may have strained resources. However, the timing of the initial diagnosis and the quality of the subsequent treatment for patients with rectal cancer were in no way compromised as a result.

## Figures and Tables

**Table 1:** Clinical parameters of patients.

	Control Group n = 65	COVID-19 Pandemic n = 55	*p*-Value
Age in years	63.03 (14.74)	64.98 (13.02)	0.448
Body mass index (BMI)	24.15 (3.71)	23.647 (4.87)	0.556
Sex (% male)	40 (61.5)	36 (65.5)	0.657
Rectal cancer: upper third (12–16 cm aa)	17 (26.2)	10 (18.2)	0.297
Rectal cancer: middle third (6–<12 cm aa)	32 (49.2)	28 (50.9)	0.855
Rectal cancer: lower third (<6 cm aa)	16 (24.6)	17 (30.9)	0.442
Neoadjuvant therapy	28 (43.1)	30 (54.6)	0.210
Progress before surgery	3 (4.6)	4 (7.3)	0.536
Palliative treatment	7 (10.8)	9 (16.4)	0.369
Surgery in total	58 (89.2)	43 (78.2)	0.099
Primary tumor resection	30 (46.2)	13 (23.6)	0.010
Surgical resection	51 (78.5)	36 (65.5)	0.112
Endoscopic resection	4 (6.2)	1 (1.8)	0.236
Tumor resection in total	55 (84.6)	37 (67.3)	0.025
Tumor resection ex domo	3 (4.6)	6 (10.9)	0.192
Ileus	7 (10.8)	2(3.6)	0.139
Perforation	3 (4.6)	0 (0)	0.107
Chronic inflammatory bowel diseases	5 (7.7)	3 (5.5)	0.624

Continuous data (age and body mass index (BMI)) are presented as mean values with standard deviations in brackets. Frequencies are presented as prevalences with portions in brackets. Statistical analysis by *t*-test and Chi-squared. A *p*-value < 0.05 is considered to be significant. aa: ab ano. In the COVID-19 group, two patients underwent ex domo primary resection and one patient showed progression with peritoneal carcinomatosis during planned primary resection.

**Table 2:** Characteristics of the operated patients.

		Control Group n = 58	COVID-19 Pandemic n = 43	*p*-Value
ASA classification	ASA1	8 (13.8)	7 (16.3)	0.728
	ASA2	28 (48.3)	22 (51.2)	0.774
	ASA3	20 (34.5)	13 (30.2)	0.652
	ASA4	2 (3.4)	1 (2.3)	0.742
Laparoscopic/robotic		42 (72.4)	35 (81.4)	0.294
Conversion		2 (4.8)	2 (5.7)	0.851
Stoma creation		57 (98.3)	41 (95.4)	0.392
		n = 51	n = 36	
Discontinuity resection for tumor resections		10 (19.6)	10 (27.8)	0.372
Clavien Dindo classification: tumor resections	0	23 (45.1)	23 (63.9)	0.084
	I	4 (7.8)	0	0.085
	II	3 (5.9)	0	0.139
	III	18 (35.3)	8 (22.2)	0.190
	IV	3 (5.9)	4 (11.1)	0.377
	V	0	1 (2.8)	0.231

Frequencies are presented as prevalences with portions in brackets. ASA = American Society of Anesthesiologists [25]. Postoperative complications were classified according to Clavien Dindo [26] (I: derivation of the normal; II: pharmacological treatment; III: surgical/interventional therapy; IV: life-threatening therapy; V: death). Statistical analysis by Chi-squared. A *p*-value < 0.05 is considered to be significant.

**Table 3:** Tumor staging.

	At the Time of Diagnosis			After Surgical/Endoscopic Tumor Resection		
	Control Group n = 63	COVID-19 Pandemic n = 55	*p*-Value	Control Group n = 54	COVID-19 Pandemic n = 37	*p*-Value
0	5 (7.9)	1 (1.8)	0.131	3 (5.6)	5 (13.5)	0.188
I	8 (12.7)	4 (7.3)	0.331	16 (29.6)	11 (29.7)	0.992
IIA	8 (12.7)	12 (21.8)	0.188	12 (22.2)	4 (10.8)	0.160
IIB	5 (7.9)	0	0.033	1 (1.9)	1 (2.7)	0.786
IIC	3 (4.8)	2 (3.6)	0.762	0	0	
IIIA	1 (1.6)	2 (3.6)	0.481	2 (3.7)	2 (5.4)	0.697
IIIB	13 (20.6)	14 (25.5)	0.534	5 (9.3)	7 (18.9)	0.181
IIIC	3 (4.8)	4 (7.3)	0.565	5 (9.3)	1 (2.7)	0.216
IV	17 (27.0)	16 (29.1)	0.799	10 (18.5)	6 (16.2)	0.777
	n = 45	n = 41				
EMVI+	19 (42.2)	19 (46.3)	0.701			
	n = 49	n = 47				
CRM+	23 (46.9)	27 (57.4)	0.303			

According to the 8th American Joint Committee on Cancer (AJCC)/Union Internationale Contre le Cancer (UICC) [27,28,29]. Extramural vascular infiltration (EMVI) and circumferential resection margin (CRM) determined by MRI [30,31]. Frequencies are presented as prevalences with portions in brackets. Statistical analysis by Chi-squared. A *p*-value < 0.05 is considered to be significant.

**Table 4:** Pathological findings.

		Control Group n = 65	COVID-19 Pandemic n = 55	*p*-Value
Grading				
	G1 & G2	54 (83.1)	50 (90.9)	0.209
	G2 & G3	11 (16.9)	5 (9.1)	
MMR		n = 45	n = 40	
	pMMR	44 (97.8)	40 (100.0)	0.343
	dMMR	1 (2.2)	0	

Mismatch repair (MMR); deficient (dMMR); proficient (pMMR). Frequencies are presented as prevalences with portions in brackets. Statistical analysis by Chi-squared. A *p*-value < 0.05 is considered to be significant.

**Table 5:** Characteristics of patients with neoadjuvant treatment.

	Clinical Stage at the Time of Diagnosis			Pathological Tumor Stage		
	Control Group n = 28	COVID-19 Pandemic n = 30	*p*-Value	Control Group n = 22	COVID-19 Pandemic n = 24	*p*-Value
Stoma before neoadjuvant therapy	15 (53.6)	10 (33.3)	0.120			
UICC stage						
0	0	0		1 (4.5)	5 (20.8)	0.101
I	0	0		6 (27.3)	4 (16.7)	0.384
IIA	4 (14.3)	3 (10.0)	0.617	4 (18.2)	2 (8.3)	0.322
IIB	3 (10.7)	0	0.066	1 (4.5)	1 (4.2)	0.950
IIC	2 (7.1)	2 (6.7)	0.943	0	0	
IIIA	0	2 (6.7)	0.164	1 (4.5)	0	0.291
IIIB	8 (28.6)	12 (40.0)	0.360	4 (18.2)	5 (20.8)	0.821
IIIC	3 (10.7)	4 (13.3)	0.760	2 (9.1)	1 (4.2)	0.499
IV	8 (28.6)	7 (23.3)	0.649	3 (13.6)	6 (25.0)	0.332
ALI				n = 22	n = 23	
				3 (13.6)	10 (43.5)	0.027
VNI				n = 22	n = 23	
				3 (13.6)	2 (8.7)	0.598
PNI				n = 14	n = 23	
				4 (28.6)	8 (34.8)	0.695

According to the 8th American Joint Committee on Cancer (AJCC)/Union Internationale Contre le Cancer (UICC) [27,28,29]. Frequencies are presented as prevalence with portions in brackets. Statistical analysis by Chi-squared. A *p*-value < 0.05 is considered to be significant. ALI: angiolymphatic invasion; VNI: venous invasion; PNI: perineural invasion. The difference between neoadjuvant pre-treated patients and resected patients results from external resection, surgical rejection, and tumor progression.

**Table 6:** Lockdown Analysis.

		Control Group n = 65	Lockdown COVID-19 n = 11	*p*-Value
T stage	T1	2 (3.1)	0	0.555
	T2	9 (13.8)	0	0.189
	T3	29 (44.6)	6 (54.5)	0.541
	T4a	12 (18.5)	1 (9.1)	0.445
	T4b	4 (6.2)	3 (27.3)	0.025
	Tx	9 (13.8)	1 (9.1)	0.666
N stage	N0	22 (33.8)	3 (27.3)	0.668
	Nx	15 (23.1)	2 (18.2)	0.719
	N+	28 (43.1)	6 (54.5)	0.479
M stage	M0	43 (66.2)	9 (81.8)	0.301
	M+	17 (26.2)	2 (18.2)	0.572
	Mx	5 (7.7)	0	0.341
		n = 63		
	0	5 (7.9)	0	0.333
UICC Stage	I	8 (12.7)	0	0.211
	IIA	8 (12.7)	3 (27.3)	0.210
	IIB	5 (7.9)	0	0.333
	IIC	3 (4.8)	1 (9.1)	0.558
	IIIA	1 (1.6)	0	0.674
	IIIB	13 (20.6)	3 (27.3)	0.622
	IIIC	3 (4.8)	2 (18.2)	0.102
	IV	17 (27.0)	2 (18.2)	0.537
Neoadjuvant treatment		28 (43.1)	8 (72.7)	0.069
Primary resection		30 (46.2)	1 (9.1)	0.021
Tumor resection in total		55 (84.6)	8 (72.7)	0.333

Comparison of initial tumor staging (TNM and UICC classification [27,28,29]) and therapy regime between COVID-19 pandemic of all lockdown periods summarized and control group before the COVID-19 pandemic. Frequencies are presented as prevalences with portions in brackets. Statistical analysis by Chi-squared. A *p*-value < 0.05 is considered to be significant. Tx/Nx/Mx showed non-evaluable results. N+/M+ showed patients with lymph node involvement and distant metastases. Lockdown times in Germany: 22 March 2020–04 May 2020; 02 November 2020–26 December 2020 and 27 December 2020–09 May 2021.

## Data Availability

The dataset generated and analyzed during the current study is available from the corresponding author upon reasonable request. All authors consent for publication.

## Supplementary Materials

The following supporting information can be downloaded at: https://www.mdpi.com/article/10.3390/jcm13123568/s1, Table S1: Tumor staging at the time of diagnosis, Table S2: Pathological tumor stage, Table S3: Characteristics of neoadjuvant-treated patients.
