# Supplementary material for: Influence of the COVID-19 Pandemic on Patients with Rectal Cancer
Source: J Clin Med. 2024 Jun 18;13(12):3568. doi: 10.3390/jcm13123568 (PMC11204882; doi:10.3390/jcm13123568)
Supplement: Supplementary file 1 [file jcm-13-03568-s001.zip › jcm-3022589-supplementary.pdf]

Table S1. Tumor staging at the time of diagnosis.

		Control Group n=65	COVID-19 Pandemic n=55	p-value
Neoadjuvant therapy		28 (43.1)	30 (54.6)	0.210
T stage	T1	2 (3.1)	1 (1.8)	0.660
	T2	9 (13.8)	6 (10.9)	0.628
	T3	29 (44.6)	27 (49.1)	0.624
	T4a	12 (18.5)	7 (12.7)	0.391
	T4b	4 (6.2)	6 (10.9)	0.348
	Tis			
	Tx	9 (13.8)	8 (14.5)	0.913
N stage	N0	22 (33.8)	14 (25.5)	0.318
	N+	28 (43.1)	29 (52.7)	0.292
	Nx	15 (23.1)	12 (21.8)	0.869
M stage	M0	43 (66.2)	37 (67.3)	0.897
	M+	17 (26.2)	16 (29.1)	0.720
	Mx	5 (7.7)	2 (3.6)	0.345

According to the 8th American Joint Committee on Cancer (AJCC)/Tumor-Node-Metastasis (TNM) staging system**.** Frequencies are presented as prevalence with portions in brackets. Statistical analysis by Chi square. A p-value <0.05 is considered to be significant. Tx/Nx/Mx/Rx showed non-evaluable results. N+/M+ showed patients with lymph node involvement and distant metastases. ALI: angiolymphatic invasion; VNI: venous invasion; PNI: perineural invasion.

Table S2. Pathological tumor stage.

		**Control Group n=55**	**COVID-19 Pandemic n=37**	**p-value**
T stage	T0	3 (5.5)	5 (13.5)	0.179
	T1	8 (14.5)	5 (13.5)	0.889
	T2	13 (23.6)	8 (21.6)	0.821
	T3	25 (45.5)	15 (40.5)	0.641
	T4a	5 (9.1)	2 (5.4)	0.513
	T4b	0	1 (2.7)	0.220
	Tis	0	1 (2.7)	0.220
	Tx	1 (1.8)	0	0.410
N stage	N0	33 (60.0)	21 (56.8)	0.757
	N1	11 (20.0)	13 (35.1)	0.105
	N2	8 (14.5)	3 (8.1)	0.351
	Nx	2 (3.6)	0	0.241
	N+	1 (1.8)	0	0.410
M stage	M0	45 (81.8)	31 (83.8)	0.8077
	M1a	8 (14.5)	5 (13.5)	0.889
	M1b	2 (3.6)	1 (2.7)	0.805
R stage	R0	52 (94.5)	36 (97.3)	0.526
	R1	1 (1.8)	1 (2.7)	0.775
	Rx	2 (3.6)	0	0.241
ALI		**n=53**	**n=36**	
		15 (28.3)	12 (33.3)	0.612
VNI		**n=53**	**n=36**	
		7 (13.2)	3 (8.3)	0.475
PNI		**n=33**	**n=35**	
		7 (21.2)	9 (25.7)	0.662

According to the 8th American Joint Committee on Cancer (AJCC)/Tumor-Node-Metastasis (TNM) staging system. Frequencies are presented as prevalence with portions in brackets. Statistical analysis by Chi square. A p-value <0.05 is considered to be significant. Tx/Nx/Mx/Rx showed non-evaluable results. N+/M+ showed patients with lymph node involvement and distant metastases. ALI: angiolymphatic invasion; VNI: venous invasion; PNI: perineural invasion.

Table S3. Characteristics of neoadjuvant treated patients.

	Clinical stage at the time of diagnosis			Pathological tumor stage		
	**Control Group n=28**	**COVID-19 Pandemic n=30**	**p-value**	**Control Group n=22**	**COVID-19 Pandemic n=24**	**p-value**
**T stage**				**yT stage**		
T0	0	0		1 (4.5)	5 (20.8)	0.101
T1	0	0		2 (9.1)	2 (8.3)	0.927
T2	1 (3.6)	3 (10.0)	0.334	6 (27.3)	2 (8.3)	0.090
T3	20 (71.4)	17 (56.7)	0.242	11 (50.0)	12 (50.0)	1.000
T4a	4 (14.3)	6 (20.0)	0.565	2 (9.1)	2 (8.3)	0.927
T4b	3 (10.7)	4 (13.3)	0.760	0	0	
Tis	0	0		0	1 (4.2)	0.333
**N stage**				**yN stage**		
N0	6 (21.4)	2 (6.7)	0.103	13 (59.1)	12 (50.0)	0.536
N+	17 (60.7)	23 (76.7)	0.189	7 (31.8)	9 (37.5)	0.686
Nx	5 (17.9)	5 (16.7)	0.905	2 (9.1)	3 (12.5)	0.711
**M stage**				**yM stage**		
M0	17 (60.7)	22 (73.3)	0.306	19 (86.4)	18 (75.0)	0.332
M+	8 (28.6)	7 (23.3)	0.649			
M1a				3 (13.6)	5 (20.8)	0.520
M1b				0	1 (4.2)	0.333
Mx	3 (10.7)	1 (3.3)	0.268			
**R stage**				**yR stage**		
R0				22 (100.0)	23 (95.8)	0.333
R1				0	1 (4.2)	0.333

According to the 8th American Joint Committee on Cancer (AJCC)/Tumor-Node-Metastasis (TNM) staging system. Frequencies are presented as prevalence with portions in brackets. Statistical analysis by Chi square. A p-value <0.05 is considered to be significant. The difference between neoadjuvant pre-treated patients and resected patients results from external resection, surgical rejection and tumor progression.
